# Three years in – changing plan features in the U.S. health insurance marketplace

**DOI:** 10.1186/s12913-018-3198-3

**Published:** 2018-06-15

**Authors:** Caitlin N. McKillop, Teresa M. Waters, Cameron M. Kaplan, Erin K. Kaplan, Michael P. Thompson, Ilana Graetz

**Affiliations:** 1 Department of Preventive Medicine, University of Tennessee Health Science Center, 66 N. Pauline St., Memphis, TN 38163 USA; 2 Economics Department, Rhodes College, 2000 North Parkway, Memphis, TN 38112 USA; 3 Present address: Department of Economics, State University of New York at Cortland, 28 Graham Ave., Cortland, NY 13045 USA; 4 Present address: Department of Health Management and Policy, University of Kentucky College of Public Health, 111 Washington Avenue, Lexington, KY 40536 USA; 5 Present address: Department of Cardiac Surgery, University of Michigan Medical School, 5331K Frankel Cardiovascular Center, 1500 E. Medical Center Dr., SPC 5864, Ann Arbor, MI 48109 USA

**Keywords:** Affordable care act, Health insurance exchanges, Silver plan premiums, Geographic variation

## Abstract

**Background:**

A central objective of recent U.S. healthcare policy reform, most notably the Affordable Care Act’s (ACA) Health Insurance Marketplace, has been to increase access to stable, affordable health insurance. However, changing market dynamics (rising premiums, changes in issuer participation and plan availability) raise significant concerns about the marketplaces’ ability to provide a stable source of healthcare for Americans that rely on them. By looking at the effect of instability on changes in the consumer choice set, we can analyze potential incentives to switch plans among price-sensitive enrollees, which can then be used to inform policy going forward.

**Methods:**

Data on health plan features for non-tobacco users in 2512 counties in 34 states participating in federally-facilitated exchanges from 2014 to 2016 was obtained from the Centers for Medicaid & Medicare Services. We examined how changes in individual plan features, including premiums, deductibles, issuers, and plan types, impact consumers who had purchased the lowest-cost silver or bronze plan in their county the previous year. We calculated the cost of staying in the same plan versus switching to another plan the following year, and analyzed how costs vary across geographic regions.

**Results:**

In most counties in 2015 and 2016 (53.7 and 68.2%, respectively), the lowest-cost silver plan from the previous year was still available, but was no longer the cheapest plan. In these counties, consumers who switched to the new lowest-cost plan would pay less in monthly premiums on average, by $51.48 and $55.01, respectively, compared to staying in the same plan. Despite potential premium savings from switching, however, the majority would still pay higher average premiums compared to the previous year, and most would face higher deductibles and an increased probability of having to change provider networks.

**Conclusion:**

While the ACA has shown promise in expanding healthcare access, continued changes in the availability and affordability of health plans are likely to result in churning and switching among enrollees, which may have negative ramifications for their health going forward. Future healthcare policy reform should aim to stabilize marketplace dynamics in order to encourage greater care continuity and limit churning.

## Background

A central objective of recent healthcare policy reform in the United States has been to make coverage more secure and to extend affordable health insurance to those who were previously uninsured or only covered intermittently [1]. The Health Insurance Marketplace, established under the Patient Protection and Affordable Care Act (ACA) in 2014, is the most notable example of such a policy initiative. However, rising premium costs, changes in plan availability, and changes in issuer participation (in particular issuers leaving the market) have been observed over the first 3 years, which raises some concerns about the stability of the Marketplace, in particular how these changes affect the health plan choices available to consumers. Adding further to the concerns about whether the ACA policy initiatives can actually achieve their stated objectives is the possibility that the healthcare law could be repealed or replaced under the current Republication administration, although legislation to repeal the ACA has stalled as of March 2017, and it is uncertain whether it will be revived.

Despite the uncertainty about the future of the ACA and the health insurance marketplaces, understanding the effect of changing market dynamics since 2014 on the choice set of plans available to consumers can offer valuable insight for the development of future policy initiatives that aim to provide health insurance coverage on a national scale. Using publicly available health plan data for all 2512 counties in the 34 states participating in the federally-facilitated insurance marketplaces we examined changes in premiums, medical deductibles, issuers and plan types, and issuer participation over time, including how the available set of health plans, and their respective characteristics, affect potential incentives to switch plans among price sensitive enrollees. In addition, we analyzed variation in the cost of staying in the same plan from the previous year versus switching to the new cheapest plan offered in the county in order to assess whether and to what extent geographic differences existed.

### Overview of the ACA marketplace and other health insurance systems

In the United States, the ACA Health Insurance Marketplace was designed to be a competitive, organized health insurance market (comprised of health insurance exchanges administered either by the federal government or by individual states) where consumers could easily comparison-shop and buy insurance based on price, benefit design, and cost-sharing, either online, by phone, or with in-person help at designated locations. Individuals with access to affordable coverage are also required to purchase health insurance or pay a penalty; those without access to affordable coverage, for whom the lowest-cost bronze plan would cost more than 8% of their income, are exempt from the penalty. In 2016, the penalty was the greater of $695 per person or 2.5% of income. There is a yearly period when individuals can enroll in a health insurance plan, or switch to a different plan. Open enrollment is also a feature of many health systems in Europe that underwent significant healthcare reform in the 1990s, including in Switzerland (biannual enrollment windows), the Netherlands (annual), Germany (first annual, now monthly), and Belgium (quarterly) [2–5].

ACA health insurance plans are designed to be actuarially equivalent, and are classified into four “metal levels” based on the percentage of health care costs that are covered for the average enrollee: bronze (60% of costs covered by insurance), silver (70%), gold (80%) and platinum (90%); under this design bronze plans, for example, have the lowest premiums, but the highest expected out-of-pocket costs. Out of pocket costs may include large deductibles (which can exceed $6000 per person for bronze plans) in addition to copayments and coinsurance. In contrast, the compulsory national health insurance plan in Belgium, for example, covers major health risks (including inpatient and long-term care) for the entire population, while in Germany a minimum benefit package is required but the insured are expected to pay co-payments for certain services, such as prescription drugs and hospital stays [6].

ACA plan premiums are established on the basis of a modified community rating system, wherein they are only allowed to vary on the basis of four factors: geographic region, family size, age, and tobacco use. Premiums for each age are based on a schedule of fixed ratios determined by a standardized age curve, such that premiums for a 64-year-old are three times greater than those for a 21-year-old; most plans follow the federally established age curve, with the exception of four states and the District of Columbia, who have established their own age curves. Variation in ACA plan features such as premiums and medical deductibles is quite large, however, and even plans with the same actuarial value are likely valued differently by individuals depending on expectations for their healthcare needs. High premium variability has also been shown in empirical studies of health systems in Europe, such as those in Belgium and Switzerland [2, 3].

### Premium subsidies

Households with incomes between 100 and 400% of the federal poverty level (FPL) are also eligible for subsidies, which are determined on the basis of income level and the premium cost of the silver plan with the second-lowest premium (the “benchmark” plan) in the county, such that the benchmark plan can be purchased with a fixed percentage of income. In a previous paper by Graetz, Kaplan and others [7], the example of an individual earning an income that was 200% of the FPL ($23,340) in 2014 was used to demonstrate subsidy calculations – this individual would be able to purchase the benchmark plan using 6.3% of their income, or $123 per month. For a benchmark plan with a monthly premium of $300, the subsidy would be equal to the difference in this market cost of the plan and the amount the individual would pay per month based on the fixed income threshold, or $177 per month. Similar calculations can be performed for any income between 100 and 400% of the FPL, with the exception of the case where a benchmark plan has a premium cost that is at or below the income threshold ($123 in the previous example), where no subsidy would be received (see Graetz, Kaplan et al. 2014 for additional details on subsidy calculations). It is also important to note that the subsidy received can be used to purchase any plan in the Marketplace, including the lowest-cost silver plan and the less expensive bronze plans. Approximately 19 and 20% of consumers enrolled in lowest-cost silver plans and bronze plans, respectively, in each year of the Marketplace.

### Changes in the marketplace

Understanding how Marketplace stability affects the affordability of health plans and the choices that consumers have (including how relative costs of plans impact incentives to switch), is of particular importance. In a previous study, we analyzed the impact of rising premiums on affordability [8]. In this study, we focused our analysis on the effect of changing market dynamics on the set of health plans (and their respective characteristics, in particular their relative costs) available to individuals seeking low-cost healthcare.

## Methods

### Data

We obtained publicly available data on the characteristics of health plans and plan availability (including issuers and plan types) for non-tobacco users in all 2512 counties in the 34 states participating in the federally-facilitated exchanges for 2014–2016 from the Centers for Medicaid & Medicare Services (CMS). Tobacco users were excluded from this analysis since they may face up to a 50% increase in premiums due to the tobacco surcharges. We used crosswalk files provided by CMS to link plans between 2014 and 2015 and between 2015 and 2016 in order to examine how specific plan features changed from year to year as well as to determine which plans were discontinued [https://data.healthcare.gov]. Counties were chosen as the unit of analysis since rating areas are generally based on county lines.

Although we cannot actually observe enrollment choices at the county level or for specific plans within a metal tier level due to data limitations, we can observe the set of plans, and their respective characteristics, that are available to consumers. Individuals facing reenrollment decisions for plans that continued the following year were also affected by rising premiums: over half of silver plans that had been the lowest-cost initially (in 2014 or 2015) were no longer the cheapest option in the subsequent year (2015 or 2016). These consumers had to consider whether to stay in their current plan and possibly face higher premiums or switch to a different plan. By looking at changes in the consumer choice set, we can analyze potential incentives to switch plans.

The primary analysis in this paper centers on silver plans, since they are the most popular. Due to the absence of county-level enrollment data, however, we also examined changes in bronze plan features in order to provide the most representative picture of market stability (see Appendix for bronze plan results). We chose to specifically focus on the lowest-cost silver and bronze plans for two main reasons. First, since consumers tend to prioritize monthly premiums over other plan features when choosing a health insurance plan [9, 10], the lowest-cost bronze and silver plans were likely appealing entry-level and middle-level plans for enrollees who qualify for premium and cost-sharing subsidies. Thus, we expected these individuals to be more price sensitive (i.e. most likely to be affected by the trend of rising premiums) than those enrolled in gold or platinum plans, and consequently more likely to need to switch plans to maintain affordable coverage. Second, nearly 70% of all purchased Marketplace plans have been silver plans in each year since the exchanges were established [11–13]. This is partly due to many low-income consumers being eligible for cost-sharing reductions (discounts applied to deductibles, copayments, and coinsurance), but only if they enroll in silver plans [14, 15].

We analyzed how changes in individual plan premiums and other plan features affected incentives for consumers aged 27, 50, and 60 who faced a reenrollment decision, including both subsidy-eligible (incomes at or below 400% of the FPL) and non-subsidy-eligible (incomes over 400% of the FPL) individuals. These specific ages were chosen to provide a representative picture of the impact on consumers in different age ranges, which is important since age is one of the four factors by which insurance premiums can vary.

### Cost of staying

Since we were not able to observe actual stay vs. switch decisions, we considered the potential reenrollment options that consumers in the lowest-cost silver plan could encounter in the subsequent year. Such consumers would face one of three possible scenarios – their original plan would: (1) remain the lowest-cost silver plan in the county; (2) be offered, but no longer be the lowest-cost; or (3) be discontinued. Individuals enrolled in 2014 or 2015 plans who did not actively select a new plan were, by default, enrolled in the linked 2015 or 2016 plan, while those enrolled in a plan that was discontinued were automatically disenrolled and would not have coverage unless they actively selected a new plan. “Active” re-enrollees are individuals who returned to the Marketplace to select a new plan or to actively renew their existing plan [14].

Using available health plan data, we constructed a series of “scenarios” facing consumers. For both sets of years (2014–15, 2015–16), we identified the lowest-cost silver plan in the baseline year (2014 or 2015) for each county. We then determined the status of each plan the following year (2015 or 2016) and the subsequent impact on hypothetical reenrollment decisions. We categorized plans that were discontinued as “Switch Only,” since individuals enrolled in those plans would have to choose a new plan to maintain coverage. Plans that remained the lowest-cost option were categorized as “Stay Only,” since there would be no premium savings by switching plans; while these individuals could still face higher year-over-year premiums, their plan was still the cheapest compared to all other silver plans in the county. The changes in plan features for the “Stay Only” and “Switch Only” plans are shown in Table 1, along with the number of counties that would have experienced these two scenarios each time period. For plans that were no longer the cheapest option, consumers could either stay in the same plan (“Choice to Stay”) or save money in monthly premiums by switching to the new lowest-cost plan (“Choice to Switch”). The changes in plan features for this third scenario (for both the “Choice to Stay” and “Choice to Switch” options) are shown in Table 2, along with the number of counties that would experienced this scenario each time period. Finally, the cost of staying was calculated by taking the difference in the after-subsidy premium cost between staying in the same plan or switching to the new lowest-cost plan. Cost of staying results are shown in Table 3 for subsidy-eligible individuals (using 300% of the FPL as an example) and those not eligible for subsidies (> 400% of the FPL), and for all three potential scenarios. As long as an enrollee is receiving a subsidy, the incremental cost of staying or switching would be the same; we confirmed this by conducting additional robustness calculations for subsidy-eligible income levels at 200 and 400% FPL (not shown in the table for simplification purposes).

**Table 1:** Changes to lowest-cost silver plan features: “Stay Only” and “Switch Only”

	2014–2015	2015–2016	2014–2016
Stay Only	*N = 987 (39.3%)*	*N = 641 (25.5%)*	*N = 365 (14.5%)*
Premiums			
Avg. Year-Over-Year Change (%)	7.7%	13.3%	18.4%
Avg. Year-Over-Year Change ($)	$41.00	$76.50	$105.77
Counties with Premium Increase (%)	90.3%	92.2%	95.1%
Counties with Premium Decrease (%)	9.7%	7.8%	4.9%
Medical Deductible			
Avg. Year-Over-Year Change ($)	-$1.82	-$148.36	-$234.04
Avg. Year-Over-Year Change (%)	−0.1%	−4.1%	−5.9%
Issuer/Plan Type			
Counties with No Change (%)	99.9%^†^	90.6%^††^	60.5%
	2014-2015	2015–2016	2014–2016
Switch Only	*N = 175 (7.0%)*	*N = 159 (6.3%)*	*N = 352 (14.0%)*
Premiums			
Avg. Year-Over-Year Change (%)	23.3%	21.5%	25.7%
Avg. Year-Over-Year Change ($)	$120.43	$110.88	$133.15
Counties with Premium Increase (%)	93.1%	100.0%	98.0%
Counties with Premium Decrease (%)	6.9%	0.0%	2.0%
Medical Deductible			
Avg. Year-Over-Year Change ($)	$1271.71	$133.33	$525.43
Avg. Year-Over-Year Change (%)	52.5%	4.4%	20.3%
Issuer/Plan Type			
Counties with No Change (%)	0.0%	0.0%	0.0%

^†^For those who stay in the 2014 plan, the % of counties that did not retain the same issuer and plan type is due to one lowest-cost silver plan type changing from an HMO to a PPO in one county in Georgia

^††^For those who stay in the 2015 plan, the % of counties that did not retain the same issuer and plan type is due to Aetna Health Inc. acquiring Coventry Health Care Inc. in counties in Iowa (40) and North Carolina (13). Members did not experience significant changes in care, and all provider locations remained open after the change. The issuer and plan type serve as a proxy for provider network. Further, there were 10 counties in Pennsylvania where the issuer was different in the following year, based on the information from the crosswalk file used to link 2015 and 2016 plans

**Table 2:** Changes to lowest-cost silver plan features: “Choice to Stay” vs. “Choice to Switch”

	2014–2015 *N = 1350 (53.7%)*		2015–2016 *N = 1712 (68.2%)*		2014–2016 *N = 1795 (71.5%)*	
	Choice to Stay	Choice to Switch	Choice to Stay	Choice to Switch	Choice to Stay	Choice to Switch
Premiums						
Avg. Year-Over-Year Change (%)	7.1%	−1.5%	19.9%	10.7%	27.4%	13.5%
Avg. Year-Over-Year Change ($)	$42.31	-$9.18	$118.09	$63.08	$156.37	$77.08
Counties with Premium Increase (%)	87.2%	49.9%	97.0%	81.5%	95.3%	82.0%
Counties with Premium Decrease (%)	12.8%	50.1%	3.0%	18.5%	4.7%	18.0%
Medical Deductible						
Avg. Year-Over-Year Change ($)	$52.52	-$42.52	-$515.01	$369.93	-$541.67	$347.21
Avg. Year-Over-Year Change (%)	1.6%	−1.3%	−14.3%	10.3%	−15.1%	9.7%
Issuer/Plan Type						
Counties with No Change (%)	99.1%	25.1%	88.3%	36.8%	36.2%	5.1%

**Table 3:** Changes in after-subsidy premiums for lowest-cost silver plans

With Subsidy at 300% FPL						
	2014–2015			2015–2016		
	Change in Premium		Cost of Staying^a^	Change in Premium		Cost of Staying^a^
	*Stay*	*Switch*	*Difference*	*Stay*	*Switch*	*Difference*
Remained Lowest-Cost	*N = 987 counties*			*N = 641 counties*		
27-year-old	$14.51		$0.00	$19.73		$0.00
50-year-old	$8.07		$0.00	-$0.44		$0.00
60-year-old	$9.33		$0.00	-$0.80		$0.00
No Longer Lowest-Cost	*N = 1350 counties*			*N = 1712 counties*		
27-year-old	$19.04	-$1.00	$20.04	$38.98	$17.55	$21.43
50-year-old	$37.10	$3.11	$33.99	$36.37	$0.04	$36.33
60-year-old	$54.89	$3.41	$51.48	$54.95	-$0.06	$55.01
Discontinued	*N = 175 counties*			*N = 159 counties*		
27-year-old		$46.50	No Coverage		$37.04	No Coverage
50-year-old		-$1.17	No Coverage		$2.27	No Coverage
60-year-old		-$3.06	No Coverage		$1.98	No Coverage
No Subsidy at > 400% FPL						
	2014–2015			2015–2016		
	Change in Premium		Cost of Staying	Change in Premium		Cost of Staying
	*Stay*	*Switch*	*Difference*	*Stay*	*Switch*	*Difference*
Remained Lowest-Cost	*N = 987 counties*			*N = 641 counties*		
27-year-old	$15.84		$0.00	$29.54		$0.00
50-year-old	$26.99		$0.00	$50.34		$0.00
60-year-old	$41.00		$0.00	$76.50		$0.00
No Longer Lowest-Cost	*N = 1350 counties*			*N = 1712 counties*		
27-year-old	$16.50	-$3.54	$20.04	$45.89	$24.46	$21.43
50-year-old	$27.95	-$6.04	$33.99	$77.90	$41.58	$36.33
60-year-old	$42.31	-$9.18	$51.49	$118.09	$63.08	$55.01
Discontinued	*N = 175 counties*			*N = 159 counties*		
27-year-old		$46.50	No Coverage		$43.64	No Coverage
50-year-old		$79.25	No Coverage		$73.51	No Coverage
60-year-old		$120.43	No Coverage		$110.88	No Coverage

^a^Premium levels shown for nonusers of tobacco only. Cost of staying is the difference in the change in monthly after-subsidy silver premium between the linked plan and the lowest-cost option. For those who switched, both the silver plans were lowest-cost. For those who stayed, only the 2014 or 2015 silver plan was lowest-cost. Finally, in counties where the silver plan was discontinued, if enrollees did not actively select a new plan, they would no longer have coverage. Calculations were the same between 2015 and 2016

### Analysis

To examine geographic variation in the cost of staying in the same plan in both sets of years (2014 to 2015, 2015 to 2016), we created maps showing the cost of staying in the same plan year-over-year for every county in the 34 states participating in the federally-facilitated exchanges. The maps represent cost of staying for a 60-year-old at 300% of the FPL; as noted above, findings are similar across income levels for subsidy-eligible individuals, and given the fact that the impact of changes to older individuals is greater due to the structure of the age curves, we chose a 60-year-old as the example to present more interesting results. All maps were generated using QGIS, version 2.0.1 (Creative Commons). To illustrate changes in plan features at the county level we summarized changes in premiums, medical deductible amounts, issuers and plan types; all computations were conducted using Stata software, version 13.1 (StataCorp), and were performed for each age (27, 50, and 60) and income level, for all counties in the 34 states with federally-facilitated exchanges, and for both sets of years, unless otherwise specified.

## Results

### Geographic variation in the cost of staying

**Fig. 1:**
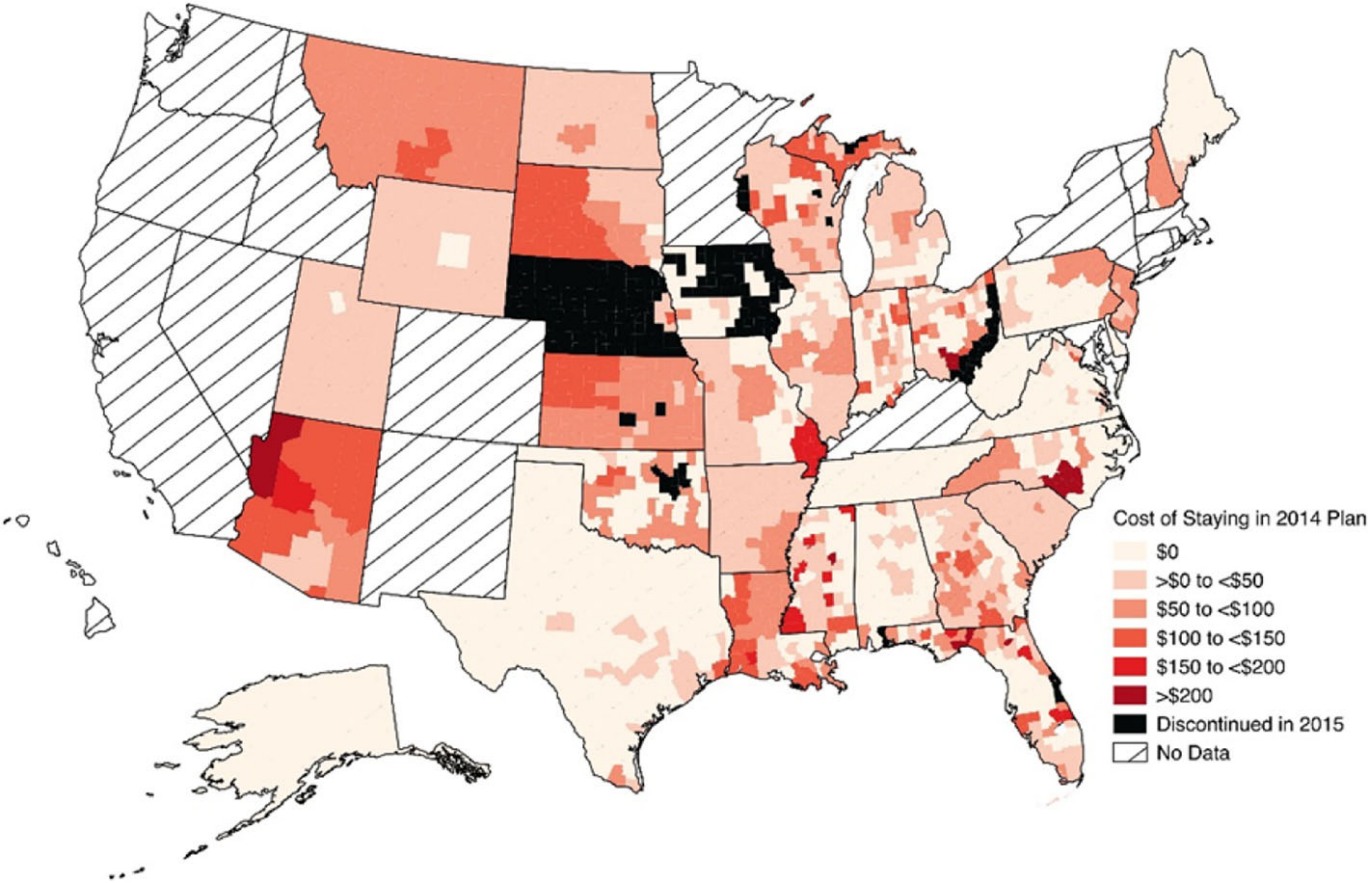
Geographic Variation in the Cost of Staying in a Silver Plan: 2014–2015. Areas with diagonal lines, labeled “No Data,” represent states that did not participate in federally-facilitated exchanges (i.e. have state marketplaces). The cost of staying was calculated as the difference between: the change between the monthly after-subsidy premium of the 2014 silver plan and the linked 2015 plan, and the change between the 2014 plan and the lowest-cost silver plan in 2015. Black represents counties where the 2014 lowest-cost silver plan was discontinued in 2015. Computed costs of staying apply to a 60-year-old

**Fig. 2:**
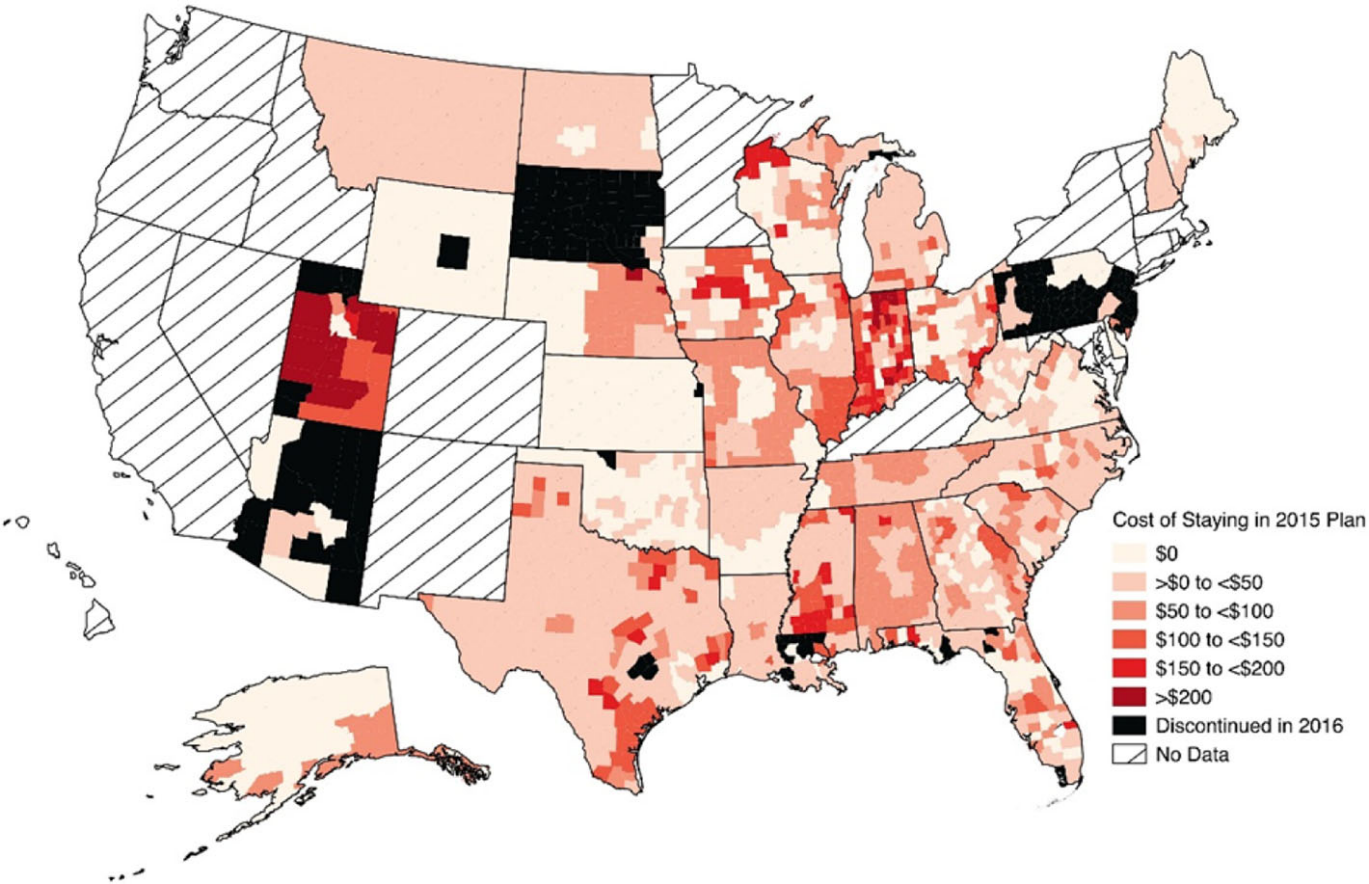
Geographic Variation in the Cost of Staying in a Silver Plan: 2015–2016. Areas with diagonal lines, labeled “No Data,” represent states that did not participate in federally-facilitated exchanges (i.e. have state marketplaces). The cost of staying was calculated as the difference between: the change between the monthly after-subsidy premium of the 2015 silver plan and the linked 2016 plan, and the change between the 2015 plan and the lowest-cost silver plan in 2016. Black represents counties where the 2015 lowest-cost silver plan was discontinued in 2016. Computed costs of staying apply to a 60-year-old

Figure 1 and Fig. 2 show geographic variation in the cost of staying in the same plan versus switching to a different plan in 2015 or 2016 for consumers previously enrolled in the lowest-cost silver plan. The lightest areas represent the 39.3 and 25.5% of counties where the lowest-cost silver plan remained the lowest-cost in 2015 and 2016, respectively. Since consumers enrolled in these plans had no cheaper option in terms of premiums when facing reenrollment, there was no cost ($0) to staying in the same plan. It is important to note that a $0 cost to staying does not necessarily mean that the plan’s year-over-year premium did not change, just that switching to any other silver plan in the county would not save the consumer any money. The graduated colors of light pink to dark red indicate that in most counties in 2015 (53.7%) and 2016 (68.2%), the previous year’s lowest-cost silver plan was still available, but no longer offered the lowest premium. In these counties, the average monthly premium cost of staying for a 60-year-old was $51.48 ($0.00–$315.14) in 2015, and $55.01 ($0–$322.60) in 2016. In the remaining counties (7.0% in 2015 and 6.3% in 2016), the lowest-cost silver plan was discontinued (black). Although we only show the cost of staying for a 60-year-old at 300% FPL, the geographic pattern was similar across all ages and income levels (both subsidy-eligible at below 400% FPL, and non-subsidy-eligible at greater than 400%) for both sets of years.

The average cost of staying is also shown in Table 3 for both subsidy-eligible and non-subsidy-eligible consumers. For plans that remained the lowest-cost, the cost of staying was $0, since all other plans offered were more expensive in comparison, while the “cost” for individuals whose plan was discontinued was no coverage the following year. In counties where the lowest-cost silver plan from the previous year was still available, but was no longer the cheapest, the cost of staying in 2015 and 2016 was an additional $51.48 and $55.01 in monthly premiums, on average.

### Changes in silver plan features and availability

Table 1 shows changes in silver plan features for the “Stay Only” and “Switch Only” scenarios, while changes for consumers faced with either “Choice to Stay” or “Choice to Switch” are shown in Table 2. In general, although average premiums for the lowest-cost silver plans increased on average between 2014 and 2015, they rose by approximately the same percentage (7.7 and 7.1%, respectively) regardless of whether that plan remained the cheapest option or not. However, between 2015 and 2016, not only did average premiums increase by a larger percentage than in the previous time period, premiums for silver plans that were no longer the cheapest increased by significantly more (19.9%) than those that remained the cheapest option (13.3%) year-over-year.

#### “Stay Only” and “Switch Only” Plans

Table 1 indicates that consumers almost always had a higher premium year-over-year regardless of whether their initial silver plan remained the lowest-cost or if their plan was discontinued. For instance, over the three-year time period average monthly premiums for plans that remained the cheapest increased by 18.4% and premiums became higher in 95.1% of counties, while premiums for residents of counties where their 2014 was discontinued by 2016 increased by 25.7% on average, even under the assumption that these consumers switched to the new lowest-cost silver plan in the county. However, opposite effects were observed for the medical deductibles: while individuals whose plan was discontinued faced an average increase in their annual deductible of $525.43 between 2014 and 2016, those whose plan remained the cheapest actually experienced a decrease of $234.04. In contrast, individuals with employer-sponsored plans experienced an increase in their annual deductible between 2014 and 2016 (by approximately $261, on average), in addition to increases in premiums [16, 17].

Thus, for consumers whose plan remained the cheapest, rising premiums may not always indicate a worsening situation – those with concurrent decreases in their deductible may have been better off from these changes. However, by 2016 silver plans remained the lowest-cost in all 3 years in only 14.5% of counties. Overall, only 1.6% of counties had plans that remained the cheapest and had decreases in deductibles between 2014 and 2016. For consumers whose plans were discontinued, the average year-over-year change in deductible for 2015–2016 was much less than the change observed for 2014–2015 ($133.33 vs. $1271.71, respectively). However, the fact remains that these consumers still faced increases in both premiums and deductibles over the 3 years.

#### *“Choice to Stay”* vs. *“Choice to Switch” Plans*

Table 2 indicates that in both sets of years more than half of silver plans that were lowest-cost initially were no longer the cheapest option in the subsequent year (53.7% of counties in 2015, and 68.2% in 2016). Further, over the entire three-year time period, almost three-quarters of lowest-cost silver plans that continued to be offered were no longer the cheapest option (71.% of counties from 2014 to 2016). Enrollees in these plans had the option to (1) stay in the plan or (2) switch to a new plan.

#### *2014–2015*

Consumers whose 2014 silver plan was no longer the lowest-cost and who chose to stay in that plan would encounter a year-over-year increase in premiums of 7.1% on average, and premium increases occurred in 87.2% of counties. However, by switching to the new lowest-cost plan, these individuals could save money in average monthly premiums both comparatively and year-over-year (see Table 3), and would have a slightly lower deductible (decrease of $42.52 annually). Despite the potential premium savings, consumers who switched would also likely have to change providers or networks: in only 25.1% of counties would the new lowest-cost silver plan have the same issuer and plan type as the consumer’s 2014 plan.

#### *2015–2016*

Consumers whose 2015 silver plan was no longer the lowest-cost and who chose to stay in that plan would encounter a year-over-year increase in premiums of 19.9% on average, a substantially higher increase compared to the previous time period; further, premium increases occurred in almost all counties (97.0%). In addition, while consumers who chose to switch to the new lowest-cost silver plan in 2016 could save money relative to staying in their 2015 plan (see Table 3), they would still face an average 10.7% increase in premiums relative to what they paid in 2015, and, unlike 2015, would experience an average increase in their annual deductible of approximately $370. The increased likelihood of having to switch providers or networks remained high, but was somewhat less of a potential issue compared to the previous time period: the issuer and plan type remained the same upon switching in 36.8% of counties in 2016.

#### *2014–2016*

Over the three-year time period, average premiums for silver plans that were lowest-cost in 2014 but were not in 2016 increased by 27.4%, while those that remained the lowest-cost option increased by 18.4%. Overall, even if consumers switched to the cheapest plan in both sets of years, they would still encounter an average increase of 13.5% in premiums and a change of approximately $350 in their average deductible (in only 11.0% of counties did plans that continued but were no longer the cheapest have decreases in deductibles between 2014 and 2016). Additionally, the issuer and plan type were only the same in 5.1% of counties.

## Discussion

Between 2014 and 2016, average Marketplace health plan premiums increased across most of the country [18]. Premiums for the lowest-cost silver plan increased by approximately 3.0% in 2015, and 10.5% in 2016 [19]. Additionally, in 7.0 and 6.3% of counties the lowest-cost silver plan was discontinued the following year (2015 and 2016, respectively), in many cases due to an issuer exiting the market entirely. These changes in plan availability led to disruption in coverage for many consumers [20]; such individuals would actively need to choose a new plan in order to maintain health insurance coverage. Some evidence for churning (i.e. plan switching) does exist at the state level (the most specific level of data that is publicly available): among consumers in a 2014 plan that continued to be offered on the federally-facilitated exchanges in 2015, approximately 53% were active re-enrollees, and of those, more than half (54%) switched plans [12]. These proportions increased to approximately 70 and 61%, respectively, in 2016 [13].

We analyzed changes in health plan features and plan availability over the first 3 years of the Health Insurance Marketplace, and found that in over half of all counties the lowest-cost silver plan was no longer the cheapest option the following year, and only 14.5% remained the lowest cost after 2 years (from 2014 to 2016). Although the majority of consumers could save money by switching, they would still pay higher average premiums year-over-year, and most would face higher deductibles and an increased probability of having to change provider networks. Even though specific cost-sharing features of plans may change year-over-year, since Marketplace metal levels are defined by their actuarial value (the average share of health costs covered taking into account all plan features such as deductibles and copayments), the average out-of-pocket costs for most should be about the same for all plans within a metal level [15]. There may be additional concerns regarding disruption in coverage for consumers due to issuers exiting the Marketplace (about 6–7% of issuers of lowest-cost silver plans left the market each year).

### Continued premium growth

Our analysis found that in counties where a different silver plan became the new cheapest option in 2015, the average premium of that new lowest-cost silver plan decreased slightly across years. In contrast, increases in average premiums were observed for all silver plans that continued in 2016, not just for those that were no longer the lowest-cost (Table 2). This suggests there were new entrants to the market that drove down prices in 2015 but not in 2016, since premiums increased steadily regardless of whether the plan remained the lowest-cost. Overall, even if consumers switched to the cheapest plan in both sets of years, they would still encounter an average increase of 13.5% in premiums between 2014 and 2016. Further, the 7.7% increase in premiums in 2015 was nearly double that of employer-sponsored plans in 2015, and the 13.3% increase in 2016 was more than four times that of employer-sponsored plans in 2016 [16, 21]. This suggests that a number of plans were underpriced in the first year of the Marketplace. In fact, according to the Congressional Budget Office (CBO), 2014 ACA premiums for the benchmark plan were approximately 15% lower than expected [22], thus, the pattern of increasing premiums could at least in part be due to the market correcting for the initial too low pricing strategies by insurers.

A more in-depth exploration into the dynamics of competition in the Marketplace is a possible direction for future work, such as analyses related to insurers pricing strategies. For instance, a new lowest-cost plan in the market could be the result of market competition working efficiently such that the new plan represents some type of equilibrium, or the result of an insurer entering the market with the objective of underbidding all other insurers in an effort to gain market share. It’s likely that more detailed financial data on insurers and more years of data of the existence of the Marketplace would be needed in order to determine which case, if either, was the correct one.

### Plan discontinuation

Although the percentage of counties where the issuer of the lowest-cost silver plan left the market in 2015 or 2016 was fairly small (~ 7.5%), in both sets of years our analysis indicated substantially higher premiums and deductibles for the new lowest-cost silver plan in these counties. Issuer/plan departure from counties or entire states could result in significant disruption of coverage for consumers, since they are automatically disenrolled. The failure of several nonprofit health insurance co-ops is one reason for issuer exit from the Marketplace; by 2016, more than half of the original ACA co-ops were no longer operating due to lack of profitability [20]. Many of these co-ops that ultimately failed were approved despite warnings about weaknesses in their business plans from third-party analysts, such as Deloitte Consulting LLP [23].

### How changes in plan features may impact consumer decision-making

Although not the only consideration for consumers, plan premiums have been shown to be an important factor in decisions to purchase health insurance [9, 24, 25]; thus, rising premiums for silver plans may promote plan switching by increasing the likelihood that consumers will feel the need to comparison-shop each year to find an affordable health plan. An important implication of switching plans is the increased likelihood of having to change provider networks. Results from our analysis indicated that residents of most counties who could save money by switching plans in 2015 and 2016 would no longer have the same issuer (~ 75 and 63% of counties, respectively), which may increase the probability of having to switch providers. The ability to keep the same provider is often a key factor when choosing a plan and is associated with higher care quality [9, 10]. Thus, despite potential premium savings, frequent plan switching could limit long-term continuity of care.

On the other hand, Marketplace consumers are more likely to have been previously uninsured [26], and individuals without dependable health insurance coverage tend to have lower health literacy [9, 27, 28] and less experience navigating the health care system [28]; thus, although the option to switch plans on an annual basis is beneficial for consumers, the specific population targeted by the ACA may find the task of re-evaluating their purchase decisions year after year to be particularly challenging, which may limit plan switching. The complexity of insurance choices in addition to uncertainty about future healthcare utilization may contribute to inertia in health insurance decisions [29, 30].

Fig. 1 and Fig. 2 show that the cost of staying can vary significantly by geographic region, suggesting heterogeneity in monthly premium changes and possible geographic differences in the likelihood of switching plans. For example, in 2016, a 60-year-old living in Summit County, Utah in 2015 could save approximately $323 per month (or $3876 per year) if she switched to the new lowest-cost silver plan, whereas a 60-year-old resident in any county in Delaware would have no cost incentive to switch. Additional interpretation would benefit from adding county-level demographic and socioeconomic data (including enrollment data, if possible) to the CMS dataset, since it is possible that some other systematic factors at the county level (e.g. proportion of racial/ethnic minorities, rural vs. urban area) may influence consumers’ incentives to switch plans, the trade-offs involved or insurer’s decisions to stay or leave the marketplaces.

### Policy recommendations

There are several potentially policy-relevant implications from our findings in this paper. We chose to focus our discussion on two areas that we believe to be particularly important: (1) the burden on consumers from having to consider reenrollment decisions every year, and (2) the pattern of rising premiums. For each, we explore ideas for policy initiatives that aim to increase to the long-term stability of the Marketplace.

#### Annual reenrollment and consumer burden

Our findings point to an increased likelihood that consumers, particularly those who are price sensitive, would be compelled to comparison-shop each year to find the most affordable health plan. However, navigating the health care system to figure out which plan is most suitable, not an easy task for anyone, is likely to be that much more difficult for people who previously were uninsured. In a study involving individuals most likely to use the marketplaces, approximately two thirds of those surveyed reported gaps in their understanding of key health insurance concepts, such as deductibles, co-payments, premiums, coinsurance and provider networks; this knowledge gap was particularly high for those in the Marketplaces’ target population who were under 30, not working, in racial/ethnic minority groups, and who had incomes between 138 and 400% of the FPL (i.e. those eligible for subsidies) [31]. While personal assistance with the application and enrollment process is available, the effort and resources to provide help will only increase as more individuals enroll in the Marketplace. Providing enrollees with tailored educational materials pertaining to their plan is one potentially low-cost way to decrease the burden of having to choose the best plan each year. Further, information about consumer preferences related to health insurance could be collected using the online system for enrollment, and then used to create targeted information about which plans might be most suitable for consumers’ particular healthcare needs each year.

#### Rising premiums and insurance risk pools

Premiums that continue to increase over time pose a real threat to the long-term stability of the Marketplace, both related to retention and the ability to attract new enrollees. Insurers being unfamiliar with the needs of the Marketplace enrollees is one likely reason for the rise in premiums over the first 3 years [20, 32].

An unexpectedly high proportion of older, sicker consumers purchasing insurance in the Marketplace is another likely explanation for rising premiums. The current subsidy design makes it less expensive for older adults to purchase plans cheaper than the benchmark plan compared to younger adults [7, 8]. As a result, insurance risk pools may be skewed in the direction of a larger than expected proportion of older adults with higher healthcare needs, which then subsequently perpetuates the need for higher premiums and instability in the Marketplace. More expensive premiums may inhibit the willingness of younger adults to purchase insurance, who may instead choose to pay the penalty. The current political uncertainty about enforcement of the mandate and whether the subsidies will be maintained may further discourage healthier adults from enrolling. Adjusting the subsidy design to make purchasing insurance more attractive to younger adults is one clear way to combat the trend in rising premiums.

### Limitations

While four plan metal tiers are offered in the Marketplace, we focused our analysis on the lowest-cost silver and bronze plans. The majority of Marketplace consumers have enrolled in silver plans in each of the 3 years; additionally, there is evidence that most enrollees tend to migrate to the cheaper silver plans (i.e. the lowest and second-lowest cost plans) [33, 34]. Further, given the similarity of changes in silver plan premiums to the changes observed for plans overall [19], we might expect the general trends to be similar for other metal levels. Our results from analyzing changes to the lowest-cost bronze plans suggest this to be the case; we observed a similar trend upward in the proportion of counties where plans were no longer the cheapest (50.8 and 63.8% of counties in 2015 and 2016, respectively), and in only 11.9% of counties did the lowest-cost bronze plan remain the cheapest option all 3 years.

We also could not determine if individuals actually switched plans if their lowest-cost silver plan from the previous year was no longer the cheapest, due to limitations of the data. Instead, we determined the implications for the three potential scenarios that consumers could face upon reenrollment, in which they would have to make a choice either to stay in the same plan or switch to a new plan, and discussed the potential influence of several factors. There is evidence for substantial inertia in health insurance plan choices in general [35–40]; however, data from the Office of the Assistant Secretary for Planning and Evaluation does indicate considerable plan switching in both 2015 and 2016, at least at the state-level [12, 13]. Policymakers would benefit from future research that looks at whether actual switching at the individual level took place. It may also be beneficial to put the findings from this paper into a context with other types of markets (such as health insurance systems in European countries). Finally, because we did not have data to link state-facilitated marketplace plans between time periods, we were unable include them in our analysis.

## Conclusion

While ACA policy initiatives have shown promise in providing health insurance coverage for individuals who previously would not have been able to access or afford care, continued changes in health plan features and affordability will making staying in the same plan year-after-year costly for many. High plan switching and churning could have negative ramifications going forward. Future healthcare policy should aim to stabilize the Marketplace by making sure the mandate to purchase health insurance is enforced, by decreasing the burden to consumers of having to consider reenrollment every year (e.g. by providing targeted educational materials), and by changing the current subsidy design to encourage greater enrollment of younger, healthier adults.

## Appendix

**Table 4:** Changes to lowest-cost bronze plan features: “Stay Only” and “Switch Only”

	2014–2015	2015–2016	2014–2016
Stay Only	*N = 1100 (43.8%)*	*N = 784 (31.2%)*	*N = 300 (11.9%)*
Premiums			
Avg. Year-Over-Year Change (%)	9.4%	16.5%	26.4%
Counties with Premium Increase (%)	89.5%	98.5%	92.7%
Counties with Premium Decrease (%)	10.5%	1.5%	7.3%
Medical Deductible			
Avg. Year-Over-Year Change ($)	-$25.14	$178.64	$197.83
Avg. Year-Over-Year Change (%)	−0.4%	3.4%	3.5%
Issuer/Plan Type			
Counties with No Change (%)	99.2%^†^	82.3%^††^	39.7%
	2014-2015	2015–2016	2014–2016
Switch Only	*N = 136 (5.4%)*	*N = 126 (5.0%)*	*N = 334 (13.3%)*
Premiums			
Avg. Year-Over-Year Change (%)	21.2%	23.1%	30.5%
Counties with Premium Increase (%)	95.6%	100.0%	100.0%
Counties with Premium Decrease (%)	4.4%	0.0%	0.0%
Medical Deductible			
Avg. Year-Over-Year Change ($)	$1408.46	$361.90	$665.42
Avg. Year-Over-Year Change (%)	31.5%	10.0%	15.9%
Issuer/Plan Type			
Counties with No Change (%)	1.5%	0.0%	0.01%

^†^For those who stay in the 2014 plan, the % of counties that did not retain the same issuer and plan type is due to HealthSpan Integrated Care taking over coverage from Kaiser Foundation Health Plan of Ohio in seven Ohio counties

^††^For those who stay in the 2015 plan, the % of counties that did not retain the same issuer and plan type is due to Aetna Health Inc. acquiring Coventry Health Care Inc. in counties in Iowa (99), North Carolina (24), South Carolina (1) and Georgia (21). Members did not experience significant changes in care, and all provider locations remained open after the change. The issuer and plan type serve as a proxy for provider network

**Table 5:** Changes to lowest-cost bronze plan features: “Choice to Stay” vs. “Choice to Switch”

	2014–2015 *N = 1275 (50.8%)*		2015–2016 *N = 1602 (63.8%)*		2014–2016 *N = 1877 (74.8%)*	
	Choice to Stay	Choice to Switch	Choice to Stay	Choice to Switch	Choice to Stay	Choice to Switch
Premiums						
Avg. Year-Over-Year Change (%)	9.3%	−0.2%	23.7%	15.5%	33.5%	20.3%
Counties with Premium Increase (%)	90.7%	55.5%	99.4%	90.3%	97.2%	85.9%
Counties with Premium Decrease (%)	9.3%	44.5%	0.6%	9.7%	2.8%	14.1%
Medical Deductible						
Avg. Year-Over-Year Change ($)	-$0.98	$353.49	$163.62	$213.05	$132.01	$457.88
Avg. Year-Over-Year Change (%)	0.1%	8.6%	3.4%	6.2%	2.9%	11.3%
Issuer/Plan Type						
Counties with No Change (%)	98.7%	20.0%	95.3%	44.4%	32.9%	11.9%

**Table 6:** Changes in after-subsidy premiums for lowest-cost bronze plans

With Subsidy at 300% FPL						
	2014–2015			2015–2016		
	Change in Premium		Cost of Staying^a^	Change in Premium		Cost of Staying^a^
	*Stay*	*Switch*	*Difference*	*Stay*	*Switch*	*Difference*
Remained Lowest-Cost	*N = 1100 counties*			*N = 784 counties*		
27-year-old	$13.30		$0.00	$19.71		$0.00
50-year-old	$4.14		$0.00	$2.36		$0.00
60-year-old	$4.11		$0.00	$3.99		$0.00
No Longer Lowest-Cost	*N = 1275 counties*			*N = 1602 counties*		
27-year-old	$16.75	-$0.74	$17.49	$36.09	$20.79	$15.30
50-year-old	$35.22	$5.64	$29.58	$30.27	$4.22	$26.05
60-year-old	$50.81	$6.07	$44.74	$45.88	$6.47	$39.41
Discontinued	*N = 136 counties*			*N = 126 counties*		
27-year-old		$33.61	No Coverage		$31.23	No Coverage
50-year-old		-$34.38	No Coverage		-$11.24	No Coverage
60-year-old		-$53.35	No Coverage		-$19.68	No Coverage
No Subsidy at > 400% FPL						
	2014–2015			2015–2016		
	Change in Premium		Cost of Staying	Change in Premium		Cost of Staying
	*Stay*	*Switch*	*Difference*	*Stay*	*Switch*	*Difference*
Remained Lowest-Cost	*N = 1100 counties*			*N = 784 counties*		
27-year-old	$13.86		$0.00	$29.05		$0.00
50-year-old	$23.58		$0.00	$49.37		$0.00
60-year-old	$35.81		$0.00	$74.90		$0.00
No Longer Lowest-Cost	*N = 1275 counties*			*N = 1602 counties*		
27-year-old	$15.35	-$2.14	$17.49	$42.95	$27.65	$15.30
50-year-old	$25.93	-$3.65	$29.58	$73.08	$47.04	$26.04
60-year-old	$39.20	-$5.54	$44.74	$110.96	$71.42	$39.54
Discontinued	*N = 136 counties*			*N = 126 counties*		
27-year-old		$33.33	No Coverage		$38.10	No Coverage
50-year-old		$56.81	No Coverage		$64.43	No Coverage
60-year-old		$86.32	No Coverage		$97.42	No Coverage

^a^Premium levels shown for nonusers of tobacco only. Cost of staying is the difference in the change in monthly after-subsidy bronze premium between the linked plan and the lowest-cost option. For those who switched, both the bronze plans were lowest-cost. For those who stayed, only the 2014 or 2015 bronze plan was lowest-cost. Finally, in counties where the bronze plan was discontinued, if enrollees did not actively select a new plan, they would no longer have coverage. Calculations were the same between 2015 and 2016

## Abbreviations

ACA: Affordable Care Act
CBO: Congressional Budget Office
CMS: Centers for Medicaid & Medicare Services
FPL: Federal poverty level
